# Supplementary material for: Chromothripsis during telomere crisis is independent of NHEJ, and consistent with a replicative origin
Source: Genome Res. 2019 May;29(5):737–49. doi: 10.1101/gr.240705.118 (PMC6499312; doi:10.1101/gr.240705.118)
Supplement: Supplemental Material [file supp_gr.240705.118_Supplemental_file_1.zip › contigs/annotated_contigs/DB45/contig.2.DB45_length_526_mean_cov_6.50380228137.docx]

**DB45_length_526_mean_cov_6.50380228137**

```
TCTATGGTTTGAGTCAACTTGATATGATTTAAAGAAACAGAAATGCTTCCTCTCTGCCTGCAAATGACAAAACTCCCATATGCATCCAT
 >chrX:46143149-46143363 - E=4e-117
TTTGGGGGGTTAAAGCTTTAGAAGGCAGTTAGAGTGCAACTATACCAAATGAGTTCCTTTTTTTTGAGACAGGAGTCTGGCTCTGTCGC

CCAGGCTGGAGTGCAGTGGCGCGATCTCGGCTCA|CT|TGTAAAGCTCTAAGTCTGGAATACTAATTCAAGGTAGTGGGTTAGAAACTC
                                   >chrX:46142867-46143183 + E=9e-121
ATTTGGTGGCTGGGCGCGGTGGCTCACGCCTGTAATCCCAGCACTTTGGGAGGCCGAGGCAGGCGGATCACGAGGTCAGGAGATTGAGA

CCATCTTGGCTAACACGGTGAAGCCCCATCTCTACTAAAAATACAAAAATTAGCTGGGCGTGGTGGCAGGTGCCTGTAGTCCCAGCTAC

TCGGGAGGCTGAGGCAGGAGAATAGCTTGAACCCTGGATGCGGAGGTTGCAGTGAGTGAGATTGCGCCACTGCACTCCAGCCT
```
